# Editorial: Manipulation of gut microbiota as a key target to intervene on the onset and progression of digestive system diseases

**DOI:** 10.3389/fmed.2022.999005

**Published:** 2022-08-29

**Authors:** Ding Shi, Silvia Turroni, Lan Gong, Wenrui Wu, Howard Chi Ho Yim

**Affiliations:** ^1^State Key Laboratory for Diagnosis and Treatment of Infectious Diseases, National Clinical Research Center for Infectious Diseases, Collaborative Innovation Center for Diagnosis and Treatment of Infectious Diseases, Department of Infectious Diseases, The First Affiliated Hospital, Zhejiang University School of Medicine, Hangzhou, China; ^2^Jinan Microecological Biomedicine Shandong Laboratory, Jinan, China; ^3^Unit of Microbiome Science and Biotechnology, Department of Pharmacy and Biotechnology, University of Bologna, Bologna, Italy; ^4^Microbiome Research Centre, St George and Sutherland Clinical Campus, School of Clinical Medicine, University of New South Wales, Sydney, NSW, Australia

**Keywords:** gut microbiota, digestive system diseases, fecal microbiota transplantation, treatment, prognosis

It has been well acknowledged that the gut microbiota (GM), the collection mainly of bacteria that symbiotically inhabit the gut, acts as an “invisible organ” exerting profound impacts on the physiology and pathology of the gastrointestinal tract and beyond (1). In the specific context of intestinal and liver diseases, both endogenous and exogenous influences could disrupt the intestinal barrier, increasing intestinal permeability and contributing to the establishment and perpetuation of GM dysbiosis (2). Such a disturbance can result in the translocation of bacteria, bacterial-derived components (lipopolysaccharides, peptidoglycan, DNA, etc.), and bacterial metabolites from the “leaky gut” to the systemic circulation, with mechanistic implications in a variety of extra-intestinal diseases (2, 3). Therefore, researchers have proposed that gut microbes could be used as biomarkers for diagnosing diseases and predicting prognosis (4, 5). Besides, an increasing number of studies are exploring the therapeutic potential of GM manipulation. Current methods for regulating gut microecology include dietary intervention, probiotics, prebiotics, synbiotics, antibiotics, fecal microbiota transplantation (FMT), postbiotics and even genetically engineered bacteria, although for most of them there is no evidence of long-term efficacy and safety based on clinical trials. So far, dietary intervention has been successfully tested in metabolic-associated liver disorders, such as non-alcoholic fatty liver disease (NAFLD) (6). On the other hand, probiotics and prebiotics are widely used as intestinal microecological regulators in clinical practice. Probiotics are effective in the treatment of ulcerative colitis (UC), *Clostridioides difficile* infection (CDI), NAFLD, cirrhosis, and its complication, hepatic encephalopathy (HE) (7–11). Regarding the clinical treatment of the latter, the non-absorbable antibiotic rifaximin has also been widely used due to its benefits in effectively preventing the recurrence of dominant HE (12, 13). In addition, rifaximin has been proven to improve liver enzymes and endotoxemia in NAFLD and non-alcoholic steatosis hepatitis (NASH) patients (14, 15). FMT as arguably the most direct means of manipulating GM has been reported as meaningful intervention in animal experiments and some clinical applications, mainly CDI, but also UC, irritable bowel syndrome (IBS), chronic hepatitis B, alcoholic liver disease (ALD), NAFLD, liver cirrhosis, and HE (16–19). However, large randomized controlled trials are still needed to validate its implication in terms of long-term efficacy and safety. Another emerging method that precisely targets GM is bacteria that are genetically engineered to deliver bioactive molecules or express certain functionalities. Based on their activity, they may improve bacterial colonization, immune regulation, toxin metabolism, and anti-pathogen colonization by targeting relevant genes (18).

In this scenario, this special issue appeared highly topical and attracted great attention. In particular, our Research Topic aimed to discuss whether and how modulating GM by different methods could become an integral part of the prevention and treatment of digestive system diseases. In this special collection, we published original research, providing novel insights into the possible causal relationships between GM and digestive system diseases, as well as reviews specifically focused on certain disorders, namely Crohn's disease (CD) and alcohol-associated liver disease.

As anticipated above, GM has a profound and sometimes crucial influence on the health of the gut and the entire human body. With reference to intestinal diseases, Rashed et al. reviewed GM dysbiosis in CD, and enumerated the different approaches available to modulate GM as a whole (meaning bacteria, archaea, fungi, and viruses), including antibiotics, probiotics, prebiotics, synbiotics, personalized diets, and FMT. In another review, Watanabe and Kamada focused on the pathophysiological mechanisms of CD with intestinal fibrosis, which is a critical determinant of prognosis. They summarized current knowledge on the link between intestinal fibrosis in CD and GM, and discussed some GM-dependent animal models that demonstrated causality. As for colorectal cancer (CRC), previous studies have shown differences in GM communities throughout the various stages of the disease; however, the drivers of GM imbalances and how they contribute to the onset of CRC remain to be determined. In this context, Shaw et al. found that the composition of the mucosa-associated microbiota is influenced by body mass index (BMI) status in CRC patients. In particular, having a BMI >25 kg/m^2^ was associated with an overabundance of *Prevotella* and Fusobacteria, as well as an increase in trans-phylum relationships (i.e., co-occurrences and co-exclusions). Approaches to manipulate GM towards a healthier (eubiotic) profile can therefore hold great promise in treating digestive system diseases. Among them, FMT from healthy donors is one of the most effective strategies for replacing the GM of patients with various diseases. In this regard, Amorim et al. refined the gut decontamination protocols prior to FMT engraftment. Their results showed that 7 days of broad-spectrum antibiotic treatment followed by 3 weekly doses of FMT provide a simple, reliable, and cost-effective methodology for FMT in animal research.

Due to the close anatomical and functional interaction of the gut-liver axis, it is not surprising that GM also plays a crucial role in the onset and progression of liver disease. Interestingly, GM alterations may already be present at the early stage of liver damage. In particular, Sheng et al. found that GM structure and function in males with hyperuricemia and elevated levels of liver enzymes were significantly different from those in healthy individuals. Such alterations included the enrichment of pathways involved in the production of 5-aminoimidazole ribonucleotide, aromatic amino acids, and chorismate, and the depletion of pathways involved in the synthesis of beneficial metabolites, i.e., short-chain fatty acids, as well as producing taxa (e.g., *Roseburia, Ruminococcus* and *Butyricimonas* species). Changes in GM have also been observed in patients with alcohol-induced liver dysfunction. Jiao et al. identified an underrepresentation of *Faecalibacterium prausnitzii* and *Roseburia hominis*, as well as four metabolic pathways (associated with galacturonate and glucuronate catabolism, β-D-glucuronide degradation, D-galacturonate degradation, and mixed acid fermentation) in the drinking case group compared to the non-drinking case group. The changed bacterial species have been proven to be anti-inflammatory and protect liver function. Administering probiotics and/or changing dietary patterns to reverse GM dysbiosis may therefore improve liver function of alcohol drinkers. Recently, advances have also been made in elucidating the interaction between GM and alcohol-associated liver disease (ALD). Chen et al. reviewed the mechanisms by which gut bacteria and fungi contribute to the onset and development of ALD and proposed some effective treatments to restore GM, including probiotics, FMT, and specific bacteriophages.

Liver cirrhosis is the end-stage of many chronic liver diseases. GM dysbiosis is likely to occur gradually during its development but again, evidence of GM involvement and the usefulness of its manipulation is still scant. In this context, Triantos et al. discussed the risks of variceal bleeding in cirrhotic patients and found that anti-endotoxin antibody and TGF-β levels were significant predictors. Moreover, fatty acid-binding protein 2 (FABP2), a marker of enterocyte damage, was associated with 6-week mortality, suggesting that impaired intestinal barrier (potentially affected by GM) and subsequent microbial translocation may be crucial for the prognosis of liver cirrhosis patients. Therefore, GM-modulating interventions may be promising strategies for preventing/treating liver diseases. Lu et al. combined GM profiling through shotgun metagenomics with targeted metabolomics to uncover the role of lactitol supplementation in regulating GM dysbiosis and metabolic dysregulation in cirrhotic patients. The relative abundance of some health-promoting lactic acid bacteria (i.e., lactobacilli and bifidobacteria) increased, while the proportions of the pathogen *Klebsiella pneumoniae* and associated antibiotic-resistant genes/virulence factors decreased after lactitol intervention. Finally, hepatocellular carcinoma (HCC) typically develops as a result of persistent and long-term chronic liver injures followed by progression to severe fibrosis and cirrhosis. The presence of microvascular invasion (MVI) is a critical indicator of long-term survival and tumor recurrence. Zhang et al. observed significant differentiation in GM composition and structure between the HCC-MVI group and HCC patients without vascular invasion. Based on key GM signatures, a non-invasive HCC-MVI microbial prediction model was constructed using 20 bacterial families with an area under the curve (AUC) value of 94.81%.

In conclusion, the modulation of unbalanced GMs as an adjunct therapy for digestive system diseases is attracting increasing attention. Diet, probiotics, prebiotics, synbiotics, antibiotics, FMT, postbiotics and genetically engineered bacteria have been shown as potential treatment strategies for digestive and liver diseases. However, these treatments are still controversial in terms of efficacy, safety, and mechanisms of action, and must necessarily be confirmed in clinical trials with a large sample size. Nevertheless, it is still an interesting, challenging, and promising research field.

## Author contributions

All authors listed have made a substantial, direct, and intellectual contribution to the work and approved it for publication.

## Conflict of interest

The authors declare that the research was conducted in the absence of any commercial or financial relationships that could be construed as a potential conflict of interest.

## Publisher's note

All claims expressed in this article are solely those of the authors and do not necessarily represent those of their affiliated organizations, or those of the publisher, the editors and the reviewers. Any product that may be evaluated in this article, or claim that may be made by its manufacturer, is not guaranteed or endorsed by the publisher.
